# From Short- to Long-Term Effects of C-Section Delivery on Microbiome Establishment and Host Health

**DOI:** 10.3390/microorganisms9102122

**Published:** 2021-10-09

**Authors:** David Ríos-Covian, Philippe Langella, Rebeca Martín

**Affiliations:** Micalis Institute, Paris-Saclay University, INRAE, AgroParisTech, 78350 Jouy-en-Josas, France; david.rios-covian@inrae.fr (D.R.-C.); philippe.langella@inrae.fr (P.L.)

**Keywords:** cesarean section, primo-colonization, microbiota

## Abstract

The establishment of gut microbiota has been proven to be impacted by several factors during pregnancy, delivery, and neonate periods. The body of evidence describing C-section delivery (CSD) as one of the most disruptive events during early life has expanded in recent years, concluding that CSD results in a drastic change in microbiota establishment patterns. When comparing the gut microbiota composition of CSD babies with vaginally delivered (VD) babies, the former show a microbiome that closely resembles that found in the environment and the mother’s skin, while VD babies show a microbiome more similar to the vaginal microbiome. Although these alterations of normal gut microbiota establishment tend to disappear during the first months of life, they still affect host health in the mid–long term since CSD has been correlated with a higher risk of early life infections and non-transmissible diseases, such as inflammatory diseases, allergies, and metabolic diseases. In recent years, this phenomenon has also been studied in other mammals, shedding light on the mechanisms involved in the effects of a CSD on host health. In addition, strategies to revert the disruptions in gut microbiomes caused by a CSD are currently in the process of development and evaluation. In this review, we discuss the recent advances in CSD research, from the alteration of gut microbiota establishment to the possible effects on host health during early life and development.

## 1. Introduction

Delivery by cesarean section (C-section, CS) is a life saver in certain risk-related situations, such as antepartum hemorrhages, fetal distress, abnormal fetal presentation, and hypertensive disease, although the prevalence of maternal mortality and morbidity is higher after delivery by CS than after vaginal delivery (VD) [1]. The number of CSs in 2015 doubled in comparison to those registered in 2000. Furthermore, the percentage of CS deliveries (CSDs) relative to total births fluctuates depending on the regional zone—from 44.3% in Latin America and the Caribbean to 4.1% in central Africa [2,3]. In addition, CS is performed in higher rates in private medical practices than in the public sector, possibly due to economic reasons [3]. To address the current situation, the World Health Organization has established recommendations for the application of CSs, indicating that they should not exceed 10–15% of the deliveries [4]. Regarding the infant, short-term effects have been reported, including an alteration to immune development; an increase in the risk of allergies, atopy, and asthma; and different microbiota colonization patterns. In the long term, CSs have been associated with obesity and asthma in children [1,5,6,7,8,9]. Several authors have observed the vertical transmission of bacterial strains from mother to infant, this transfer being specific to each mother–child pair [10]. This vertical transmission could be altered by several neonatal factors that introduce abnormal microbiota in the newborn, a situation known as dysbiosis. CSD has been described as one of the major disruptions in microbiota colonization patterns [11], and these differences may be correlated with other health issues observed in later stages in human life. In this review, we will discuss the importance of the first colonization (primo-colonization) and its effects on human development. We will also discuss the changes induced by CSDs in the neonate microbiota and the physiological effects induced in both the mother and the baby, including a mechanistic glimpse provided by a few of the first animal models used to study this phenomenon. Lastly, we will discuss the interventions and ongoing studies focusing on correcting the “disruptive” colonization that occurs after a CSD.

## 2. Acquisition of the First Microbiota: When and Where?

### 2.1. Acquisition of the First Microbiota. Primo-Colonization and the “Sterile Womb” Controversy

As the first interaction with microbiota is traditionally considered to be during delivery, the method of delivery has been associated with different patterns of colonization; in particular, CSs have been considered as the cause of disruptions during the normal colonization of microbiota in the infant gut. Nevertheless, the possible existence of uterus microbiota may affect how we understand the process of primo-colonization as the moment and process by which the first microbiota is acquired. This moment determines the way by which host–microbiota interactions are established and, hence, how they can be studied and understood by the scientific community. The existence of a sterile environment before delivery has been debated by scientists. As a consequence of the development of NGS technologies, the sterility of the meconium and the placenta before delivery has been questioned several times, as some authors have discovered bacterial DNA in meconium samples [12]. Initial studies pointed to a very limited colonization of microbiota in meconium, as the number of bacteria found in first-pass meconium samples from vaginally delivered babies was very low in the first 24 h of life [13]. Although some authors have found low amounts of bacterial DNA in samples of placenta, meconium, and amniotic liquid, several physiological and critical aspects, such as materno-fetal barriers, immunological barriers, methodological considerations, and the possibility of obtaining germ-free animals from aseptic hysterectomies, should be considered in order to make any assumptions [12]. It is important to highlight that sampling methods have also been assessed by some authors; for example, a study including placenta samples from 20 term and preterm deliveries found bacterial DNA in the samples, but after analyzing the sequences, the differences with the negative controls were not significant [14]. In another study, bacteria were found in fetal membranes and umbilical cords but never inside the placenta, supporting the hypothesis of contamination as the source of bacterial presence in studies that challenge the “sterile womb” hypothesis [15]. As for the amniotic fluid, a recent study in a bovine C-section model revealed some positives (5 out of 24), with low amounts of bacteria in the meconium, but none of the amniotic fluid samples presented different amounts of bacterial DNA compared to the negative controls [16]. In a recent study, fecal meconium was collected before antibiotic administration during breech CSDs without labor and compared to standard VDs, first-pass meconium, and stool. It was found that the bacteria in the samples were most likely skin contaminants such as *Staphylococcus epidermidis* [17]. On the other hand, some authors still defend the hypothesis that the sterility of the human fetal intestine is not absolute, but both the number and diversity of bacteria are very low [18,19]. More scientific evidence is required for the hypothesis of in utero microbial colonization, as several questions remain unanswered in this respect. For example, why do pre-delivery bacterial communities always exist in small numbers and with low richness if the environment is nutrient rich and free for newcomers to colonize? To date, the “sterile womb” theory has enough scientific evidence and high robustness to support it, while other theories require an improved understanding and more scientific support.

### 2.2. The Concept of Primo-Colonization in the Context of Vertical Microbiota Transmission

Vertical microbiota transmission is an important concept to understand primo-colonization. As mentioned before, vertical transmission has been addressed by several authors [10,20]. One study following the microbiome and mothers over one year found that, even though the microbial species composition of babies at the beginning of their lives is similar to that of their mothers, mother and child shared the same strains, but new strains were introduced over time by environmental factors [20]. The concept of primo-colonization encompasses how the interaction between this first vertically transmitted microbiota and the host, especially in the immune system during the first weeks of life, can affect the development and maturation of the host [21]. Germ-free rats colonized by adult and suckling rat microbiota had different reactions at physiological and anatomical levels that converged almost completely when the microbiota from suckling rats evolved to a more adult microbiota [22]. In this respect, a strong immune response was observed in mice during weaning, but it disappeared when the mice were treated with antibiotics during the weaning window period. In addition, they developed a severe form of colitis, in comparison with control mice, when treated with DSS at 9 weeks of age [23]. Considering these implications of bacterial composition in the maturation of the immune system [21], it is important to investigate further to gain a more comprehensive understanding of the role that CSD might play in the development of a newborn’s immune system.

## 3. Changes in Neonate Microbiota after C-Section Delivery

One of the first approaches to the question of how delivery mode affects gut microbiota composition carried out by Dominguez-Bello et al. showed that the microbiota of vaginally delivered babies resembled their mothers’ vaginal microbiota, while CSD babies showed a composition reflecting the skin microbiota of their mothers [24]. Although this study was carried out using a small sample size, since then, several studies have investigated this phenomenon in depth, finding a reduction in *Bifidobacterium* and *Bacteroides* species as a constant sign of CSD [11,25,26]. C-section has been reported to allow the colonization of microbiota by nosocomial opportunistic bacterial pathogens, such as *Enterococcus*, *Enterobacter*, and *Klebsiella*. The differences in the microbiota were very pronounced at 4 days of life, but in infancy (>21 days), the microbiota of C-section babies and vaginally delivered babies were more similar and closely resembled maternal microbiota [11,26,27]. In addition, higher *Clostridioides difficile* carriage has been associated with CSD at 2 months of age [7]. In another study, not only did CSD babies show an enrichment of *Enterobacteriaceae* 3 days after delivery, but there was also evidence of several features of a microbiome adapted to an oxygen-rich environment [28]. When analyzing the variables that are associated with alterations in the gut microbiota composition, the delivery mode showed the highest association from 4 to 21 days of life, whereas in infancy, delivery mode and hospital site microbiome had a similar impact. These changes resulted in a reduction in *Bifidobacterium*, *Escherichia*, *Bacteroides*, and *Parabacteroides* genera, which was particularly drastic for *Bifidobacterium* and *Bacteroides* groups [11]. Studies with other cohorts also found the *Bacteroides* population depleted in CSD babies after the neonate period at 1 and 3 months, but this was not the case for the *Bifidobacterium* population. As previously described, these great structural differences also disappeared after some time and, in this case, it was at that age 6 months [7,29,30]. A reduced alpha diversity was also found in the oral microbiome in the first 6 weeks of life in CSD babies when compared to VD babies. It is important to highlight that the microbiome of different body sites in this study did not resemble the maternal body site microbiome until week 6, as the microbiome of different body sites is more homogeneous at early stages of life [27].

Mitchell et al. have proposed a new approach to understanding the establishment of gut microbiota during C-section deliveries by studying pre-labor CSDs with scheduled CSDs, of which the main difference is that post-labor CSD babies are exposed to the maternal vaginal microbiota. In this study, *Bacteroides* was present in some CSD babies during week 1, but levels were depleted in later sampling times for both CSD groups. In addition, the analysis of bacterial strain transmission between mothers and babies showed more shared strains in VD babies and mothers than in both groups of CSD babies with their respective mothers, suggesting that vaginal exposure might not be the only source of first microbiota for *Bacteroides* species [31]. The vertical transmission of strains from mother to baby was favored by VD babies in other studies, particularly affecting *Bacteroides vulgatus*, even after the neonatal period [11].

It is apparent that delivery methods could thus profoundly affect the core microbiota in a short- to mid-term manner, as several publications point to a partial recovery from 1 to 6 months, when the microbiota starts resembling an adult-like core microbiota. However, expectedly, these changes may lead to other changes in the microbiota at later stages of life. Several studies have found differences in the microbiota from 2 to 7 years of age between VD and CSD individuals [32,33,34]. According to a recent study following the microbiota of 471 Swedish infants (169 born by C-section), the alpha diversity of the microbiota from 4 months to 5 years was not affected by the mode of delivery. In contrast, 25 genera were significantly different when comparing CSD babies with VD babies [35]. The latest systematic review about the topic found higher abundances of *Bifidobacterium* and *Bacteroides* during the first 6 months of life, but for the following 6 months, *Enterobacter* and *Streptococcus* were also higher in VD infants [26]. The mother’s BMI is another factor that has an impact on the composition of microbiota: in a study comparing the microbiota acquisition of normoweight and overweight/obese women in both VD and CSD babies, researchers found differences in the microbiota acquisitions for both BMI groups in VD babies but not in the CSD group, most likely because the effect of a CSD on dysbiosis is greater than the effect of the mother’s BMI on dysbiosis [6].

The mode of delivery might not only affect the microbiome of the neonates but also the microbiota of mothers. Recent research has focused on the microbiota of the milk, specifically because intrapartum antibiotics are administered during a C-section and breastfeeding is an important factor to shape neonate microbiota [25]. The results found for milk microbiota are similar to those found for neonate gut microbiota; for example, colostrum from C-section mothers had higher amounts of skin and environmental microbiota (*Pseudomonas* spp., *Staphylococcus* spp., and *Prevotella* spp.) than mothers who delivered vaginally [36]. The origin of these bacteria remains unclear as it could be triggered by an increased permeability during a C-section; however, it could also be affected by the intrapartum antibiotics administered during the process as well as other factors, such as the absence of physiological stress or different hormonal signaling pathways during a C-section [37,38]. A deeper understanding of microbiome-specific signatures, potential developmental windows, and bacteria–host interactions is required to explain why the C-section delivery is associated with a higher risk of non-transmissible disease development.

## 4. Socio-Demographic Characteristics and Short- and Long-Term Effects of CSD in Health

CSD has been associated with an increased risk of several pathologies and revised in several meta-analysis documents (Table 1). Research-based literature is attempting to shed some light on the mechanisms behind the increased risk of disease. CSD has been associated with a higher risk of atopic sensitization, sharing several mediators with higher BMI levels in children aged between 1 and 3, including the depletion of *Bifidobacterium* and altered sIgA secretion levels [7]. It may be possible that the higher risk of comorbidity development in CSD babies is also influenced by some groups that are, apparently, unaffected by the delivery mode but behave differently because of different environmental factors. In a recent study, the differences found in the bacterial colonization patterns between CSD and VD babies aged 1 month, especially the *Staphylococcus* group, were correlated with higher weight gain at the age of 1 year for CSD babies with higher levels of *Staphylococcus*, but not in VD babies [39]. These differences have also been associated with higher BMI levels at 12 months since birth [25]. In a retrospective study of Danish children born between 1977 and 2012, it was found that in the cases of immune diseases starting at a young age, CSDs also increased the risk of asthma, systemic connective tissue disorders, juvenile arthritis, inflammatory bowel disease, immune deficiencies, and leukemia [40]. Although this study did not find any relation to coeliac disease and type 1 diabetes, a recent study with the same cohort, but with a larger sample size as the study only focused on the development of the disease independently of the debut age, found a relationship between CSD babies and these two non-transmissible diseases [41].

It is important to note that administration of intrapartum antibiotics is often part of the standard CSD process. Early breastfeeding cessation has been associated with planned CSDs, whereas a greater number of breastfeeding difficulties has been associated with emergency CSDs [50]. This is an important fact because breastfeeding may be a strategy to recover the differences in the microbiome between CSD and VD babies, as we will discuss in the next section, alongside other strategies. Nevertheless, some studies have found that the effect of a CSD is still present, even after an improvement as a result of using antibiotics as a confounding factor [45].

In vitro models are used as a mechanistic approach to understand host–microbiota crosstalk. In a study using different types of cell lines, authors found different profiles of cytokine production in TPH1 macrophage-like cell lines, presenting those treated with VD fecal water as higher in the production capacity of IL-6, IL-8, and TNF-α. Furthermore, mRNA analysis revealed a downregulation of TL4 and FOS gene expression. On the other hand, the same supernatants did not show significant differences in epithelial HT-29 cells [25].

Murine models are necessary to explore the physiological mechanisms of microbiota–host interaction, especially those in which invasive techniques are necessary. In this respect, hitherto, only a few murine models have addressed the physiological effects of CSDs on neonate development in the short- to long-term period, but in the past 5 years, the amount of evidence has increased. Different features of the immune system in CSD C56BL/6 mice have been observed, in particular a reduction in T regulatory cells and an increase in invariant NK cells, which were transferred to germ-free mice after a fecal material transfer (FMT) of CSD pups [51]. In an oxazolone colitis model, CSD mice were more sensitive to the treatment than VD mice, including higher concentrations of TNF-alpha and inflammation markers in the colon. This phenotype was transferred to germ-free mice when inoculated with CSD and VD mouse microbiota [52]. A murine model has shown social, cognitive, and anxiety deficits, both in early life and adulthood. These deficits, consisting of social recognition, maternal attachment, social novelty recognition, exaggerated anxiety behavior, and changes in the mRNA expression of the hippocampus region, corroborated part of the behaviors shown in the murine model [53]. Martinez et al. showed an increased weight gain in CSD mice compared to VD mice and the effect was more exacerbated in females [54].

One of the recently discussed possible effects of a C-section on the mother is the effect of glycosylation patterns of milk glycan epitopes [55]. Authors in this study discuss the possibility that the baby’s oral microbiota is transferred to the mother’s aureole during breastfeeding, being able to drive those differences in glycosylation, especially because CSD and VD babies carry different microbiome profiles. Further studies and a mechanistic approach need to be performed to validate this hypothesis.

## 5. Strategies to Modulate the Aberrant Microbiota of CSD Babies

One of the first strategies proposed to correct the gut microbiota of CSD babies is vaginal seeding. The exposure of CSD babies to the mothers’ vaginal fluids after birth allowed a partial restoration of the microbiota, which was more similar to the microbiota of VD babies than CSD with no treatment [56]. In contrast, a novel approach to vaginal seeding, consisting of orally feeding the baby vagina-extracted bacteria, did not show any differences between treated and control groups, with very little engraftment of maternal strains, suggesting that other sites, apart from the mother’s vagina, could be key locations of bacterial sources during delivery [57]. Nevertheless, in mice, the co-housing of CSD and VD pups partially reverted some behavioral deficits associated with CSD [53].

FMT has been successfully used in a pilot study in order to restore the microbiome of CSD babies to one that closely resembles the microbiome of VD babies [58]. It is curious that the composition of the mothers’ microbiota for an FMT was different from that found in transplanted babies, pointing to a selective outgrowth of *Bifidobacterium* and *Bacteroides* after the transplant. FMT showed to be more effective than vaginal shedding in the restoration of gut microbiota. Breastfeeding is another major factor that affects the establishment and development of gut microbiota. CSD babies that received breastfeeding showed a partial restoration of gut microbiota when compared with CSD babies that received formula feeding [59]. All these facts together open the door to encourage the use of target interventions, such as probiotics, prebiotics, and synbiotics, which are less risky than vaginal seeding or FMT [60]. Probiotics are “live microorganisms that, when administered in adequate amounts, confer a health benefit on the host”. Prebiotics are non-digestible compounds that, through their metabolization by microorganisms in the gut, modulate the composition and/or activity of gut microbiota, thus conferring a beneficial physiological effect on the host. Synbiotics are mixtures of probiotics and prebiotics that beneficially affect the host by improving the survival and implantation of live microbial dietary supplements in the gastrointestinal tract, by selectively stimulating the growth, and/or by activating the metabolism of one or a limited number of health-promoting bacteria, thus improving host welfare [61] In fact, synbiotic intervention modulated the expression of some genes lacking in C-section babies, such as the oligosaccharide metabolism [28]. In another study, delayed *Bifidobacterium* colonization was recovered with a synbiotic consisting of a mix of GOS/FOS and *B. breve* M-16V; production of acetate to levels more similar to VD babies was also recovered [62]. In the murine CSD model, treatment with a *B. breve* strain or with a prebiotic mixture of GOS/FOS partially reverted some phenotypes associated with behavioral deficits [53]. The supplementation of 10% of XOS in CSD mice partially restored the composition of gut microbiota and was able to reduce the levels of iNK cells to those found in VD mice, but this did not increase the levels of T regulatory cells [51]. Nevertheless, the importance of the *Bacteroides* group for the problems posed by CSDs point to the utilization of next-generation probiotics (NGP) or live biotherapeutic products (LBP). Till date, the use of live biotherapeutics in the modulation of CSD babies has not been published.

Other strategies to recover behavioral deficits in murine models include treatment with oxytocin, which reverted some alterations in brain development, particularly the alterations related to social deficits; however, the study lacks information about the changes produced in the gut microbiome [63].

## 6. Conclusions

As vertical transmission seems to be the predominant form of obtaining gut microbiota, with the exception of some taxa and pathogenic bacteria [10,64], the understanding of this process during the infancy period could be essential to understanding some developmental phenomena that usually occur later in life. In this respect, we know that CSD is associated with the partial loss of bacterial vertical transmission from mother to the neonate, which, over generations, could lead to the loss of key members of the core microbiota. Some authors suggest a link between the appearance of chronic diseases and the loss of ancestral microbiota, producing changes in the crosstalk between microbiota and the host immune system and, ultimately, how it is shaped later in life [65]. As previously stated, there is already enough scientific evidence to support the hypothesis of different colonization patterns during CSDs and VD babies, and that these differences tend to disappear as the microbiota mature to resemble the adult microbiota. On the other hand, some of the latest papers on this topic are corroborating the hypothesis about the multi-site origin of the first microbiota, as it seems that the vagina might not be the only place from which VD babies attain their first microbiota. For example, one study found no differences between microbiota in babies from a planned CSD and an emergency CSD, and another study showed the failure to recover the microbial composition of CSD babies by vaginal bacteria administration. This hypothesis is also supported by the improved performance of FMT over vaginal seeding in recovering the aberrant CSD microbiota. The relationship between the first contact of microbiota with the immune system and the possible long-term effects on host health still needs a more developed understanding. In this sense, several murine models have shown deficits in cognitive development as well as different effects on the maturation of the immune system, which could lead to an increased risk of non-transmissible diseases found in adults born by CSD.

## Figures and Tables

**Table 1:** Systematic reviews and meta-analysis addressing the risk of disease development associated with CSD in the last 5 years.

Readout	Type of Meta-Analysis	Sample	Outcome	Consistency	Reference
Mother and neonate general health outcomes	Literature	79 studies (29,928,274 deliveries)	Decreased risk of urinary incontinence and rectal prolapse in the mother Increased risk of asthma and obesity	Low	[5]
Cognitive outcomes	Literature	7 studies	Reduced cognitive performance (4/7)	Weak	[42]
Neurodevelopmental and psychiatric disorders	Literature	61 studies (20,607,935 deliveries)	Increased risk of autism spectrum disorder, attention-deficit/hyperactivity disorder	Med–strong	[43]
Attention-deficit/hyperactivity disorder	Literature		Small increase in attention-deficit/hyperactivity disorder	Medium	[44]
Infection-related hospitalization	Cohort study	7,174,787 deliveries (1996–2015, births from Denmark, Scotland, England, and Australia)	Higher risk in a CSD	n/a	[45]
Respiratory tract infections Asthma Type 1 diabetes Body weight	Literature	16 studies	Higher respiratory tract infections, asthma, and obesity No association with type 1 diabetes	Strong	[46]
Asthma	Literature	37 studies	Increased risk of asthma	Medium	[47]
Type 1 diabetes	Literature	9 studies (50,000,000 deliveries)	Small increase in T1D risk	Weak	[48]
					
Childhood leukemia	Literature	19 studies	Higher risk of leukemia and lymphoblastic leukemia	Weak–med	[49]

## Data Availability

Not applicable.

## Abbreviations

CSD	C-section delivery
CS	C-section
VD	Vaginal delivery
